# Reduced Birth Weight, Decreased Early-Phase Insulin Secretion, and Increased Glucose Concentrations after Oral Glucose Tolerance Test in Japanese Women Aged 20 Years with Family History of Type 2 Diabetes

**DOI:** 10.1155/2020/8822135

**Published:** 2020-12-14

**Authors:** Mari Honda, Ayaka Tsuboi, Satomi Minato-Inokawa, Kaori Kitaoka, Mika Takeuchi, Megumu Yano, Miki Kurata, Bin Wu, Tsutomu Kazumi, Keisuke Fukuo

**Affiliations:** ^1^Open Research Center for Studying of Lifestyle-Related Diseases, Mukogawa Women's University, Nishinomiya, Hyogo, Japan; ^2^Department of Health, Sports, and Nutrition, Faculty of Health and Welfare, Kobe Women's University, Kobe, Hyogo, Japan; ^3^Research Institute for Nutrition Sciences, Mukogawa Women's University, Nishinomiya, Hyogo, Japan; ^4^Department of Nutrition, Osaka City Juso Hospital, Osaka, Japan; ^5^Laboratory of Community Health and Nutrition, Department of Bioscience, Graduate School of Agriculture, Ehime University, Matsuyama, Ehime, Japan; ^6^Department of Public Health, Shiga University of Medical Science, Otsu, Shiga, Japan; ^7^Department of Food Sciences and Nutrition, School of Food Sciences and Nutrition, Mukogawa Women's University, Nishinomiya, Hyogo, Japan; ^8^Department of Endocrinology, First Affiliated Hospital of Kunming Medical University, Kunming, Yunnan, China; ^9^Department of Medicine, Kohnan Kakogawa Hospital, Kakogawa, Hyogo, Japan

## Abstract

**Introduction:**

We tested the hypothesis that family history of type 2 diabetes (FHD) is associated with reduced birth weight and reduced insulin secretion later in life.

**Materials and Methods:**

Birth weight, body composition by whole-body dual-energy X-ray absorptiometry, and homeostasis model assessment-insulin resistance were compared between Japanese women aged 20 years with positive (*n* = 73) and negative (*n* = 258) FHD. A subsample of 153 women (57 with positive FHD) underwent a 75 g oral glucose tolerance test. Multivariate logistic regression analyses were used to identify the most important determinants of FHD.

**Results:**

Women with positive as compared with negative FHD had lower birth weight (3132 ± 364 vs. 3238 ± 418 g, *p* = 0.04). However, the current fat mass index and trunk/leg fat ratio, sophisticated measures of general and abdominal fat accumulation, respectively, did not differ. Women with positive FHD had a lower insulinogenic index (2.4 ± 7.3 vs. 6.2 ± 16, *p* = 0.007) and higher area under the glucose curve (217 ± 47 vs. 198 ± 36 mg/dL/2 h, *p* = 0.006). However, fasting and postload insulinemia, homeostasis model assessment-insulin resistance, and Matsuda index did not differ. In multivariate logistic regression analysis, birth weight was marginally associated with FHD (odds ratio, 0.999; 95% confidential interval, 0.98-1.00000; *p* = 0.0509).

**Conclusions:**

FHD was associated not only with reduced birth weight but also with decreased early-phase insulin secretion and increased postload glucose concentrations in Japanese women aged 20 years. These findings may be in keeping with the fetal insulin hypothesis and provide some evidence that FHD can alter size at birth, probably through genetic and shared environmental components, which consequently resulted in decreased early-phase insulin secretion and increased glucose excursion in the early twenties. FHD was not related to sophisticated measures of general and abdominal adiposity and insulin resistance/sensitivity.

## 1. Introduction

Reduced birth weight, a widely used indicator of retarded fetal growth and intrauterine malnutrition, has been consistently associated with a higher risk of type 2 diabetes in observational studies [1–3] and meta-analyses [4–6]. Although the cause of this association is not known, it is proposed to reflect fetal programming in utero in response to maternal malnutrition in pregnancy [7]. Mendelian randomization analyses have demonstrated that genetically lowered birth weight was associated with an increased risk of type 2 diabetes [8, 9]. An alternative explanation is the fetal insulin hypothesis [10]. As insulin released from fetal pancreatic beta cells is a crucial fetal growth factor, it is suggested that genetic variants predisposing to decreased insulin secretion or action cause reduced intrauterine growth and thereby lower birth weight as well as late-onset type 2 diabetes [10]. This hypothesis assumes that insulin deficiency is already present during fetal life. Indeed, studies have shown that type 2 diabetes risk alleles, which have been shown to be associated with reduced insulin secretion and beta cell dysfunction, were related to lower birth weight [11, 12]. These observations suggest that beta cell dysfunction may already be present in prenatal life.

Family history of type 2 diabetes (FHD) is a well-known risk factor for the disease. As several studies demonstrated the familial distribution of insulin secretion and sensitivity [13–15], it seems possible that a diabetic genotype could affect fetal growth. However, studies on the association between FHD and birth weight are limited. Decreasing birth weight was associated with increased risk of type 2 diabetes within dizygotic twin pairs, suggesting that genetic factors contribute to the association between low birth weight and risk of type 2 diabetes later in life [16, 17]. In Swedish middle-aged men, an inverse association between birth weight and glucose intolerance was found in men with and without FHD [1]. These observations suggest that the association between reduced birth weight and glucose intolerance is not due to confounding from FHD. Because the prevalence of low birth weight (<2500 g) is increasing in Japan [2], because FHD was associated with lower insulin secretion rather than insulin resistance in young Japanese people [18, 19], and because there is a sex difference in birth weight (usually lower in females), we tested whether FHD was associated with not only reduced birth weight but also reduced insulin secretion in young Japanese women.

## 2. Methods

Students of the Mukogawa Women's University participated in the present study as a volunteer and consisted of female collegiate athletes and nonathletes, students of the Department of Food Sciences and Nutrition, whose details were reported elsewhere [20]. Among 481 young Japanese women reported (170 athletes and 311 nonathletes), 332 women (129 athletes and 203 nonathletes) provided FHD data whereas data on FHD were not available in 149 women. Seventy-three women were considered FHD positive (FHD+) because the questionnaire to their parents reported that a parent or a grandparent was on oral antidiabetic drugs. Unfortunately, information was not available on the extent of family history (i.e., how many family members have the condition) and the nature of the family history (paternal or maternal). Among 332 women who provided FHD data, 331 provided birth weight data. Weight at birth and height and weight at age 12 and 15 years were obtained either through maternal health check notes or child health notebook records (issued by each municipal office). There were no significant differences in anthropometric and biochemical measurements between 332 women studied and 149 women whose FHD data were not available (data not shown). Subjects with clinically diagnosed acute or chronic inflammatory diseases; endocrine, cardiovascular, hepatic, and renal diseases; hormonal contraception; and unusual dietary habits were excluded. Nobody reported receiving any medications or having regular supplements. The study was approved by the Ethics Committees of the Mukogawa Women's University (No. 07-28) to be in accordance with the Helsinki declaration. All subjects were recruited as volunteers and gave written consent after the experimental procedure had been explained.

After a 12 h overnight fast, participants underwent blood sampling, measurement of anthropometric indices, and body composition as previously described [20–22]. Among a subsample of previously reported 168 women who underwent a standard 75 g oral glucose tolerance test (OGTT) [20–22], 153 women provided FHD data (57 FHD+). The blood was withdrawn at 0 (fasting), 30 min, 1 h, and 2 h for glucose and insulin measurements. Thus, among 332 women with FHD data (73 FHD+), 153 underwent OGTT, and the remaining 179 (16 FHD+) had fasting blood samplings only. Plasma glucose (PG) was determined by the hexokinase/glucose-6-phosphate dehydrogenase method (interassay coefficient of variation (CV) < 2%). Serum insulin was measured by an ELISA method with a narrow specificity excluding des-31, des-32, and intact proinsulin (interassay CV < 6%). The area under the response curve of PG (AUCg) and serum insulin (AUCi) was calculated by the trapezoidal method. Homeostasis model assessment-insulin resistance and *β* cell function (HOMA-IR and HOMA-*β*, respectively), the Matsuda index, and the insulinogenic index (IGI) were calculated as previously reported [23–25]. The glucose disposition index (GDI) was calculated by the product of the Matsuda index and IGI.

Fat mass, bone mass, and lean mass for the arms, legs, trunk, and the total body were measured using whole-body dual-energy X-ray absorptiometry (DXA) (Hologic QDR-2000, software version 7.20D, Waltham, MA) as previously reported [26]. The leg region included the entire hip, thigh, and leg. General adiposity was assessed by the fat mass index (FMI) calculated as body fat mass in kg divided by height in meters squared. Abdominal fat accumulation was assessed by the ratio of trunk fat to leg fat [27]. Because lean mass in the arms and legs represents skeletal muscle mass, a sum of the two was used as appendicular skeletal muscle mass. Height-adjusted skeletal muscle mass was also assessed by the skeletal muscle mass index (SMI), calculated as skeletal muscle mass in kilograms divided by squared height in meters.

The data were presented as the mean ± SD. Due to deviation from normal distribution, IGI, GDI, and HOMA-IR were logarithmically transformed for analyses. The differences between two groups were compared by a *t*-test. Multivariate logistic regression analyses were done for FHD as a dependent variable. The independent variables included were those that displayed significant differences between women with and without FHD. A two-tailed value of *p* < 0.05 was considered significant. The statistics were performed with the SPSS system 17.0 (SPSS Inc., Chicago, IL).

## 3. Results

As previously reported [20–22] and shown in Tables 1 and 2, women aged 20 years were of normal weight and normoglycemic and were not insulin resistant as indicated by the mean HOMA-IR of 1.1 and 1.3. The participants consisted of 129 athletes and 203 nonathletes.

As compared with 258 women with negative FHD, 73 FHD+ had decreased birth weight, although the mean difference was small (106 g) (Table 1). However, BMI at age 12 and 15 years did not differ. Although current BMI was slightly but significantly lower in FHD+, FMI and trunk/leg fat ratio, sophisticated measures of body fat mass and distribution, did not differ. Lower SMI in FHD+ may probably be due to a lower percentage of athletes in FHD+. Nonathletic women with and without FHD did not differ in SMI (5.85 ± 0.61 and 5.84 ± 0.55 kg/m^2^, *p* = 0.9). HbA1c, fasting PG, and insulin and hence HOMA-IR and HOMA-*β* did not differ between the two groups.

**Figure 1:**
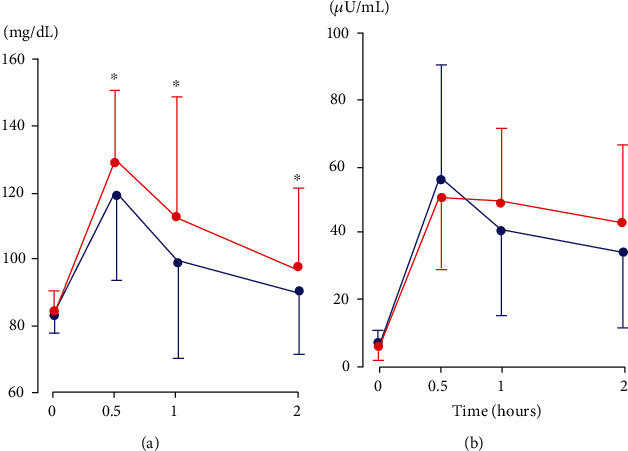
Responses of plasma glucose (a) and serum insulin (b) during a standard 75 g oral glucose tolerance test in young Japanese women with positive (red lines) and negative (blue lines) family history of type 2 diabetes. Mean ± SEM. ^∗^*p* < 0.05.

Postload PG at three time points and hence AUCg were higher in FHD+ (Table 2 and Figure 1). Although 1 h and 2 h insulin tended to be higher in FHD+, AUCi and Matsuda index did not differ. FHD+ had lower IGI and GDI. The difference in birth weight was marginally significant between women with and without FHD who underwent OGTT and provided FHD data (3139 ± 353 vs. 3235 ± 367 g, *p* = 0.056).

We have done multivariate logistic regression analyses for FHD as a dependent variable and birth weight and log IGI as independent variables. Birth weight was marginally associated with FHD (odds ratio, 0.999; 95% confidential interval, 0.98-1.00000; *p* = 0.0509). The same results were obtained on multivariate analysis which included log GDI instead of log IGI. Birth weight showed no association with variables of glucose metabolism.

## 4. Discussion

To the best of our knowledge, the present study is the first to demonstrate that FHD was associated not only with reduced birth weight but also with decreased early-phase insulin secretion and increased glucose concentrations after OGTT in Japanese women in their early twenties. FHD was not related to sophisticated measures of general and abdominal adiposity (FMI and trunk/leg fat ratio) and insulin resistance/sensitivity.

It is well known that low birth weight (<2500 g) is associated with an increased risk of developing type 2 diabetes later in life [7, 10]. The reasons underlying this association have been debated, and fetal malnutrition has been proposed as a potential mechanism (the fetal origin hypothesis) [7]. Although the proportion of female infants with birth weight < 2500 g has been reported to be increased from 4.8% in 1980 to 10.7% in 2012 in Japan [28], fetal malnutrition was not possibly the primary factor since the percentage of infants with birth weight < 2500 g was low in women with FHD (1.4%; 1/73) and in the total population (2.4%; 8/332) in the present study.

An alternative explanation is the fetal insulin hypothesis. Insulin is important for fetal growth and metabolism throughout life. The hypothesis states that common genetic factors that either increase insulin resistance or reduce insulin secretion may lead to both reduced birth weight and disease in later life [10]. Associations between reduced offspring birth weight and increased risk of fathers' risk of type 2 diabetes or insulin resistance have been reported in some [29, 30] but not in other studies [31, 32]. Associations between offspring birth weight and fathers' risk of type 2 diabetes strengthen the fetal insulin hypothesis, as fathers can only directly affect offspring fetal growth through inherited fetal genes. Genetic studies have shown that type 2 diabetes risk alleles, which have been shown to predispose to diabetes by reducing insulin secretion, were associated with reduced birth weight when inherited by the fetus [11, 12, 33, 34]. In the present study, FHD, instead of type 2 diabetes risk alleles, has been demonstrated to be associated with not only reduced birth weight but also decreased early-phase insulin secretion and increased glucose excursion in Japanese women aged 20 years. These findings are in keeping with the fetal insulin hypothesis and may provide some evidence that FHD can alter size at birth, probably through the fetal genotype, which consequently resulted in decreased early-phase insulin secretion and increased glucose excursion in Japanese women in their early twenties.

Insulin sensitivity was decreased in white children with FHD in the first decade of life, whereas there are no differences in first- or second-phase insulin secretion between children with and without FHD [35]. In contrast, it is insulin secretion that was decreased in young Japanese people with FHD whereas insulin sensitivity did not differ or rather increased in FHD youth [18, 19]. In young Japanese women in the present study, FHD was related to decreased early-phase insulin secretion but was not related to insulin resistance/sensitivity. These observations suggest that although FHD was associated with lower GDI (insulin secretion normalized for insulin sensitivity) both in white children and young Japanese people, there may be racial disparities in the underlying pathophysiology of lower GDI. It is noteworthy that GDI derived from the OGTT, which was used in the present study, provides a superior method for predicting the future development of type 2 diabetes compared with the diagnosis of impaired glucose tolerance based on the 2 h PG concentration [36].

The strengths of the present study include a homogeneous study population with scarce confounding factors as previously reported [37] and accurate and reliable measures of DXA-derived body composition. Several limitations of this study warrant consideration. The cross-sectional design of the present study complicates the drawing of causal inferences, and a single measurement of biochemical variables may be susceptible to short-term variation, which would bias the results toward the null. The recruitment procedure may also have some potential impact on the results. As the participation was voluntary, women who pay more attention to health may be more likely to participate. For example, the percentage of FHD+ was higher in women who underwent OGTT (37.3%; 57/153) compared to women with fasting blood sampling only (8.9%; 16/179, *p* < 0.001). It was also higher in nonathletes (27.1%, 55/203) compared with athletes (14.0%, 18/129, *p* = 0.005), probably because nonathletes, students of the Department of Food Sciences and Nutrition, entered the university to become nationally registered dietitians. We used crude measures of insulin sensitivity/insulin resistance and insulin secretion, which may be less accurate. Statistical power was not calculated. As we studied young Japanese women only, results may not be generalized to other genders, age populations, races, or ethnicities.

In conclusion, the present study has demonstrated that FHD was associated with not only reduced birth weight but also decreased early-phase insulin secretion associated with increased glucose concentrations after OGTT in young Japanese women. FHD was not related to sophisticated measures of general and abdominal adiposity (FMI and trunk/leg fat ratio) and insulin resistance/sensitivity. Our results are in keeping with the fetal insulin hypothesis and provide some evidence that FHD can alter size at birth, probably through genetic and shared environmental components, which consequently resulted in decreased early-phase insulin secretion and increased glucose concentrations after OGTT in Japanese women in their early twenties. If replicated, these findings may have implications for counseling and managing pregnancies to avoid adverse birth outcomes because FHD is a risk factor for gestational diabetes [38].

## Figures and Tables

**Table 1:** Characteristics of young Japanese women in the presence and absence of family history of type 2 diabetes (FHD).

	Positive FHD	Negative FHD	*p* values
	*n* = 73	*n* = 259	
Birth weight (g)	3132 ± 364	3238 ± 418	0.049
Birth weight < 2500 g (*n*, %)	1, 1.4	7, 2.7	0.442
Body mass index at age 12 (kg/m^2^)	19.0 ± 2.4	19.1 ± 2.2	0.811
Body mass index at age 15 (kg/m^2^)	20.2 ± 2.6	20.3 ± 2.1	0.747
Age (years)	20.3 ± 1.1	20.0 ± 1.2	0.079
Height (cm)	160.4 ± 6.0	161.4 ± 6.3	0.243
Weight (kg)	52.5 ± 8.5	54.9 ± 7.4	0.021
Body mass index (kg/m^2^)	20.3 ± 2.6	21.0 ± 2.1	0.023
Skeletal muscle mass index (kg/m^2^)	6.12 ± 0.75	6.39 ± 0.83	0.014
Fat mass index (kg/m^2^)	5.21 ± 1.88	5.51 ± 1.58	0.161
Trunk/leg fat ratio	1.23 ± 0.24	1.22 ± 0.24	0.689
HbA1c (%)	5.2 ± 0.2	5.2 ± 0.3	0.187
Fasting glucose (mg/dL)	84 ± 7	85 ± 7	0.497
Fasting insulin (*μ*U/mL)	5.5 ± 3.0	6.2 ± 4.5	0.216
HOMA-IR^a^	1.1 ± 0.7	1.3 ± 1.0	0.182
HOMA-*β*	106 ± 93	107 ± 73	0.914
Athletes (*n*, %)	18, 24.7	111, 42.9	0.003

Mean ± SD or *n*, %. HOMA-IR and HOMA-*β*: homeostasis model assessment-insulin resistance and *β* cell function, respectively. ^a^Logarithmically transformed.

**Table 2:** Glucose and insulin responses during 75 g oral glucose tolerance tests of young women in the presence and absence of family history of type 2 diabetes (FHD).

	Positive FHD	Negative FHD	*p* values
	*n* = 57	*n* = 96	
Fasting glucose (mg/dL)	84 ± 8	83 ± 6	0.572
2 h glucose (mg/dL)	97 ± 24	89 ± 18	0.029
Fasting insulin (*μ*U/mL)	5.6 ± 3.2	5.9 ± 3.2	0.653
2 h insulin (*μ*U/mL)	41 ± 25	34 ± 23	0.068
Matsuda index	8.9 ± 4.6	9.0 ± 3.8	0.925
Insulinogenic index^a^	2.4 ± 7.3	6.2 ± 16.1	0.007
Disposition index^a^	26 ± 87	59 ± 155	0.008
AUCg	217 ± 47	198 ± 36	0.006
AUCi	82 ± 34	77 ± 38	0.382
AUCi/AUCg	0.38 ± 0.15	0.39 ± 0.19	0.711
Athletes (*n*, %)	9, 15.8%	32, 33.3%	0.023

Mean ± SD or *n*, %. AUCg and AUCi: area under the concentration curve of plasma glucose and serum insulin, respectively. ^a^Logarithmically transformed.

## Data Availability

The ethics committee of the University does not allow us to open data except for a manuscript.
